# Unusual combination of acute aortic dissection, Mayer-Rokitansky-Küster-Hauser syndrome, and 46,XX gonadal dysgenesis: A case report

**DOI:** 10.3389/fcvm.2022.1030160

**Published:** 2022-11-10

**Authors:** Yifan Zeng, Yerong Hu, Bo Jiang, Ling Tan, Hao Tang

**Affiliations:** Department of Cardiovascular Surgery, The Second Xiangya Hospital of Central South University, Changsha, Hunan, China

**Keywords:** aortic dissection, MRKH syndrome, 46,XX gonadal dysgenesis, total aortic arch replacement, frozen elephant trunk, whole-exome sequencing

## Abstract

**Background:**

Acute Stanford type A aortic dissection (ATAAD) is a life-threatening disease. Elderly patients are the high-risk population for aortic dissection (AD). Young patients with AD usually have heritable connective tissue diseases such as Marfan syndrome and Loeys-Dietz syndrome. However, young AD patients without heritable connective tissue disease are relatively rare.

**Case presentation:**

Herein, we report a case of a 25-year-old female diagnosed with ATAAD accompanied by undeveloped secondary sexual characteristics. Computed tomography angiography (CTA) showed that her AD involved the ascending and abdominal aorta. She had undergone thoracic endovascular aortic stent graft implantation in a local hospital due to acute Stanford type B aortic dissection at age 19. No uterus or ovaries were found on CTA and transabdominal ultrasonography. Sex hormone detection revealed a low estrogen level. G-banded karyotyping analyses revealed a normal 46,XX karyotype. Finally, her abnormalities in the reproductive system were diagnosed as MRKH syndrome and 46,XX gonadal dysgenesis. Whole-exome sequencing (WES) in the patient found an SNP variant of *ACTA2* c.773G>A and *MYH11* c.5081A>G. *MYH11* c.5081A>G was also found in her mother and younger brother. Copy number variations sequencing (CNV-seq) found an approximately 109.30 Kb duplication at chromosome 6p22.3 (Chr 6: g.24920238–25029535) with a copy number of 3. We performed emergent total aortic arch replacement with frozen elephant trunk surgery, and the patient recovered well after surgery. However, her abdominal AD was stilling progression during 6 months of follow-up.

**Conclusion:**

To our knowledge, we report the world's first case of early-onset recurrent AD combined with MRKH syndrome and 46,XX gonadal dysgenesis.

## Introduction

Aortic dissection (AD) is a disease in which the intima of the aorta tear separates the intima and media of the aortic wall and allows blood to enter the false lumen. Acute Stanford type A aortic dissection (ATAAD) is a life-threatening disease with 50% mortality in the first 48 h (1). The annual incidence of ATAAD varies between 3 and 4/100,000 (2). Female incidence is lower but of higher mortality than male incidence (3).

Primary amenorrhea is a leading cause of female infertility. Mayer-Rokitansky-Küster-Hauser (MRKH) syndrome and gonadal dysgenesis (GD) are the most common causes of primary amenorrhea. Mayer-Rokitansky-Küster-Hauser syndrome, a congenital disorder also referred to as Müllerian agenesis, is characterized by congenital aplasia of the uterus, cervix, and the upper part (2/3) of the vagina in females with typical secondary sex characteristics and a normal 46,XX karyotype (4). Chromosomal regions 1q21.1, 16p11.2, 17q12, and 22q11.21 have been identified as recurrent genetic abnormalities associated with MRKH syndrome (5). Gonadal dysgenesis with female phenotype is defined as the absence or incomplete development of ovaries. Gonadal dysgenesis is mainly associated with sex chromosome abnormalities. The 45,XO, 45,X/46,XX, 45,X/46,X, dic(X), 46,XX, and 46,XY karyotypes have been reported (6). Gonadal dysgenesis with a normal 46,XX karyotype is relatively rare. The coexistence of MRKH syndrome and 46,XX GD is very rare, and only a few cases have been reported (7–9).

This article presents the world's first case of early-onset recurrent AD accompanied with MRKH syndrome and 46,XX GD.

## Case presentation

**Figure 1:**
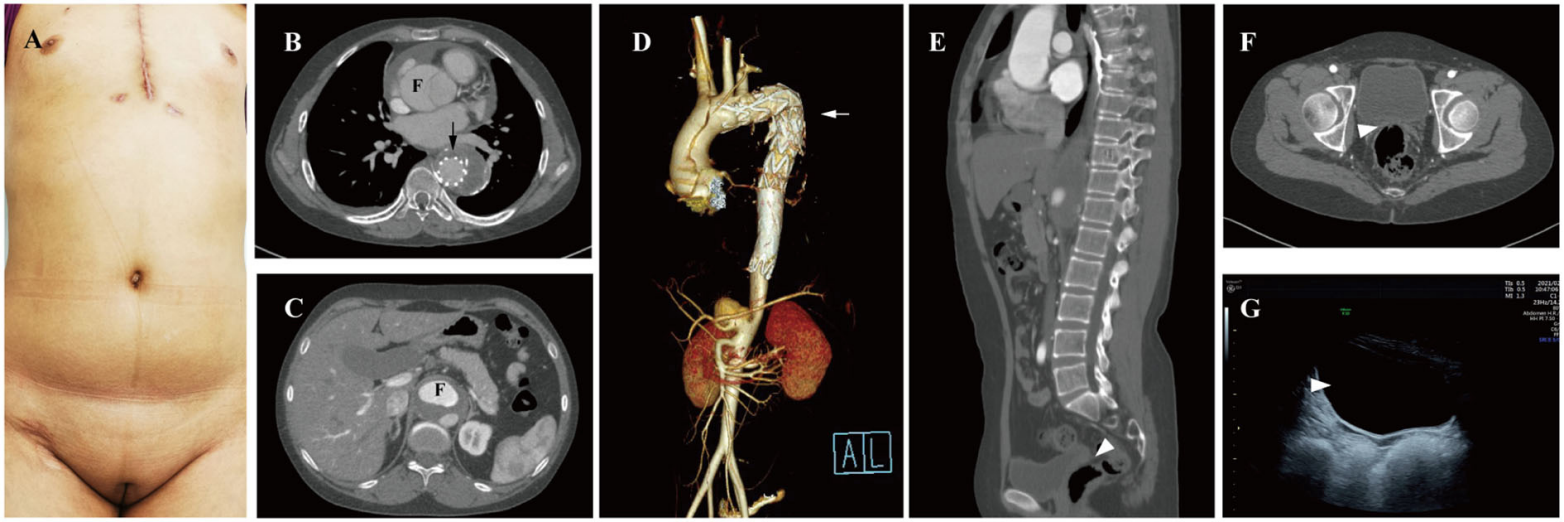
Preoperative examination of the 25-year-old female patients. **(A)** Undeveloped secondary sexual characteristics. CTA shown aortic dissection involving the ascending **(B)** and abdominal aorta **(C)**, F, false lumen. **(D)** Thoracic endovascular aortic stent was grafted (arrow); no uterus or ovaries was found between urinary bladder and rectum by CTA (**E,F**, arrowhead) and transabdominal ultrasound (**G**, arrowhead). CTA, computed tomography angiography; F, false lumen.

A 25-year-old female patient who presented with persistent chest pain was admitted to our institution. Undeveloped secondary sexual characteristics were found during physical examination (Figure 1A). Computed tomography angiography (CTA) showed that her AD involved the ascending (Figure 1B) and abdominal aorta (Figure 1C). She had undergone thoracic endovascular aortic stent graft implantation (Figures 1B,D, arrow) in a local hospital due to acute Stanford type B aortic dissection at age 19. No uterus or ovaries was found by CTA (Figures 1E,F, arrowhead) and transabdominal ultrasound (Figure 1G, arrowhead). Sexual hormone detection revealed hypergonadotropic hypogonadism (follicle-stimulating hormone 66.98 IU/L, luteinizing hormone 25.41 IU/L, and estradiol 0.1 pg/ml).

**Figure 2:**
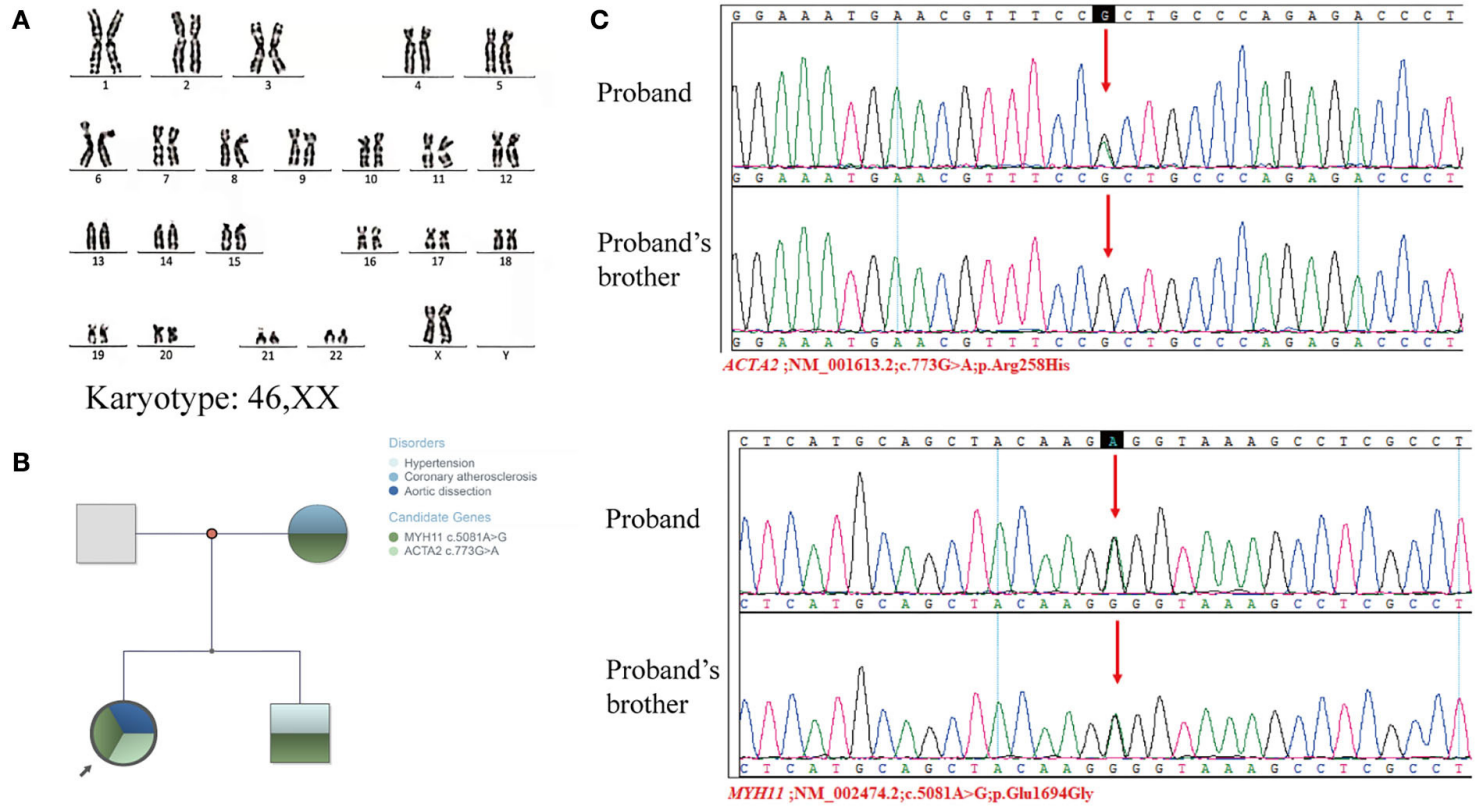
Genetic examination of the proband and proband's families. **(A)** Karyotyping analyses revealed a normal 46,XX karyotype. **(B)** Family tree. **(C)** Sanger sequencing validated the mutation of *ACTA2* c.773G>A and *MYH11* c.5081A>G in the proband and her brother.

Her height (155 cm) and weight (55 kg) were within the normal range in the Chinese female population, and she had no hypertension or family history of genetic disorders. In early pregnancy, her parents, both working at a chemical plant, had a history of exposure to potential toxicants, including cyanide, ammonia, sulfuric acid, and nitric acid. Her 18-year-old brother had hypertension, and her mother had coronary artery atherosclerosis. She presented with primary amenorrhea at age 15, was diagnosed by a gynecologist as MRKH syndrome accompanied with congenital absence of the ovaries, and was treated with hormone replacement therapy (HRT) for only 3 months. G-banded karyotyping analysis revealed a normal 46,XX karyotype (Figure 2A). The abnormalities in her reproductive system were eventually diagnosed as MRKH syndrome with 46,XX GD.

To further explore the genetic correlation between AD and congenital dysplasia of the reproductive system, we performed whole-exome sequencing (WES) and copy number variations sequencing (CNV-seq). Whole-exome sequencing of the patient and her family members, including her mother, father, and brother, showed a single nucleotide polymorphism (SNP) in the *MYH11* gene (*MYH11*; NM_002474.2; c.5081A>G; p.Glu1694Gly) and *ACTA2* gene (*ACTA2*; NM_001613.2; c.773G>A; p.Arg258His). MYH11 c.5081A>G was carried by the patient, her mother, and her brother, and ACTA2 was only carried by the patient (Figure 2B). The WES results were validated by Sanger sequencing (Figure 2C). An approximately 109-Kb (Chr 6: g.24920238-25029535) duplication at chromosome 6p22.3 was found in the patient by CNV-seq, which has not been related to the development of the uterus and ovaries. Finally, no clear genetic link between AD and abnormalities in the female reproductive system was found.

We performed emergency total aortic arch replacement and frozen elephant trunk surgery, and the patient recovered well and was discharged on postoperative day 12.

**Figure 3:**
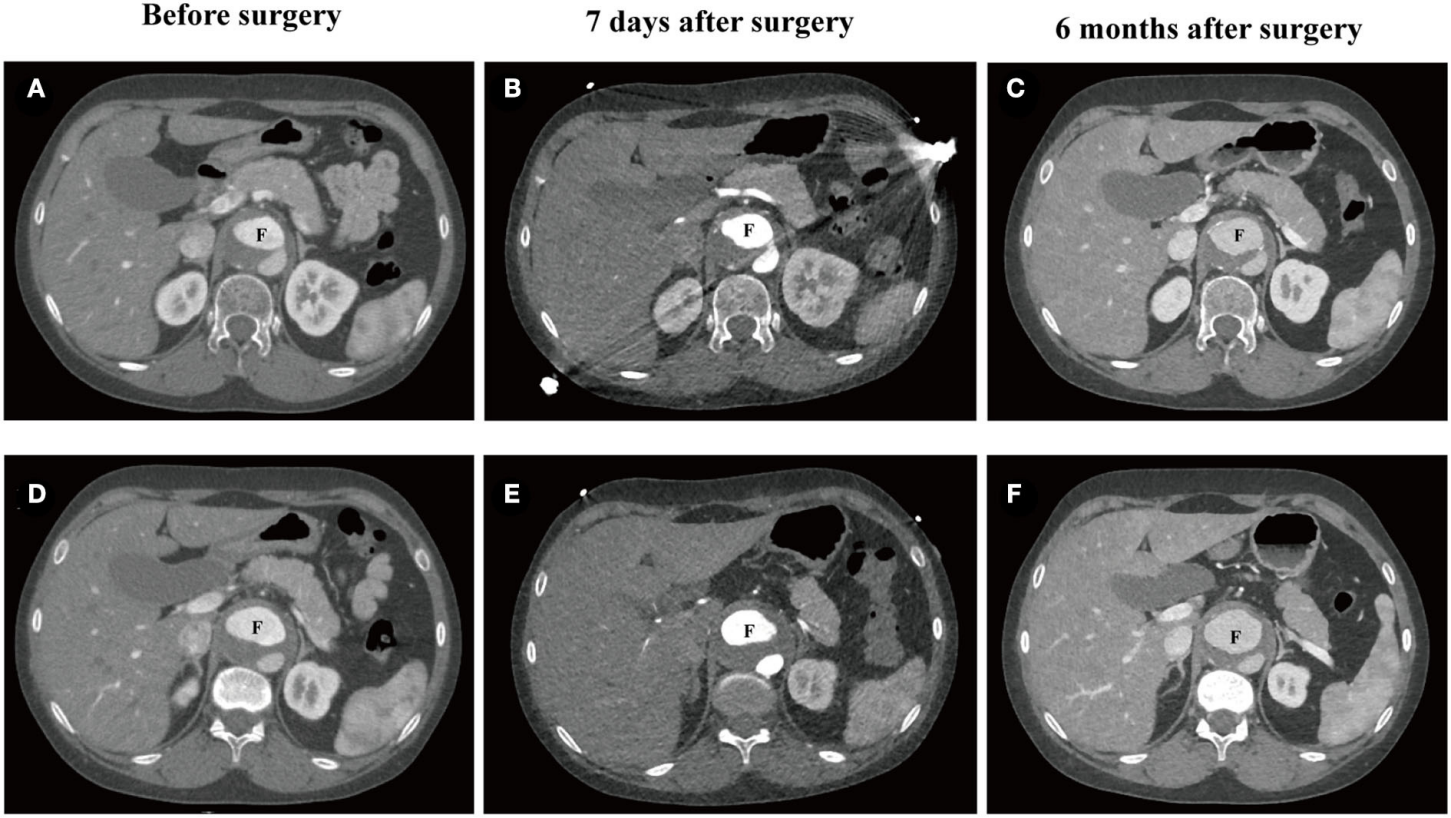
CTA of abdominal aortic dissection in the 25-year-old female patient. **(A–C)** The false lumen at the position of intimal tears before surgery (32.8 × 22.8 mm^2^), 7 days after surgery (32.6 × 22.2 mm^2^), and 6 months after surgery (35.9 × 26.8 mm^2^). **(D–F)** Maximum diameter of the false lumen of AAD before surgery (38.9 × 23.1 mm^2^), 7 days after surgery (39.3 × 24.6mm^2^), and 6 months after surgery (40.9 × 29.1 mm^2^). CTA, computed tomography angiography; F, false lumen.

The patient refused to receive HRT after surgery, and her abdominal aortic dissection (AAD) was progression during a 6-month follow-up (Figures 3A–F). The false lumen of AAD at intimal tears (Figures 3A–C) and maximum diameter (Figures 3D–F) both expanded slightly 7 days after surgery and rapidly expanded 6 months after surgery.

## Discussion

The main feature of this patient was early-onset recurrent AD accompanied with rare dysplasia of the female reproductive system (MRKH syndrome and 46,XX GD) without heritable connective tissue disease. No significant genetic relationship between AD, MRKH syndrome, and 46,XX GD was found. Nevertheless, the reason why this patient suffered from recurrent AD at a young age remains to be determined.

Single nucleotide polymorphism mutations in *MYH11* and *ACTA2* were detected in this 25-year-old female patient, suggesting that the co-mutations in *MYH11* and *ACTA2* may be involved in the pathogenesis of AD. The *ACTA2* mutation in the patient was *de novo* in her family. Besides, *ACTA2* c.773G>A has been reported to be associated with the pathogenesis of AD (10). According to the *European Reference Network for Rare Vascular Diseases (VASCERN) consensus, ACTA2* is closely related to the morbidity of cardiovascular disease, primarily seen in AD with 54% Stanford type A AD (median age 36 years) and 21% type B (median age 27 years) (11). Nevertheless, the onset age of AD in this patient was significantly earlier than the reported average age. A possible explanation is the synergistic effect of mutations in *MYH11* and *ACTA2*. A mouse experiment has shown that *Acta2*^−/−^*Myh11*^*R*247*C*/*R*247*C*^ mouse accelerates aortic enlargement and increases medial degeneration (12). However, the function of *MYH11 c*.5081A>G in cardiovascular diseases remains unclear. *MYH11* c.5081A>G is carried by the patient, her mother, and her brother (Figure 2C). Furthermore, they suffered from cardiovascular conditions. In comparison, no mutations in *MYH11* and *ACTA2* were detected in her father who had no cardiovascular diseases. Therefore, we believe that *MYH11* c.5081A>G may be essential in developing cardiovascular diseases.

Low estrogen caused by MRKH syndrome and 46,XX GD may also accelerate the progression of AD. To our knowledge, AD accompanied with gonadal anomalies is rare and mostly seen in Turner Syndrome (TS). Turner Syndrome is a genetic disorder mainly caused by complete or partial absence of one of the X chromosomes. The morbidity and mortality of TS are highly related to the presence of hypertension, aortic dilatation, aortic aneurysm, and AD (13). Hormone replacement therapy is thought to relieve aortic dilation and AD in TS patients (14). The life expectancy of TS patients after HRT is significantly prolonged, which may be related to the cardiovascular protective effects of estrogen. Besides, carotid artery intima thickness has been reported to be decreased significantly after HRT in patients with 46,XX GD and TS (15), suggesting that HRT may have a cardiovascular protective effect in patients with 46,XX GD. However, there is no clinical consensus on the necessity of using HRT in patients with AD accompanied with GD. In this case, this patient did not receive HRT after surgery, and the AAD is progressing rapidly. Therefore, we recommend using HRT for patients with estrogen deficiency. Moreover, the use of HRT in postmenopausal women with aortic aneurysm or AD still needs to be investigated.

The molecular basis of GD and MRKH syndrome is still not known. CNV-seq found a 109-Kb (Chr 6: g.24920238–25029535) duplication at chromosome 6p22.3, but no reports related to MRKH syndrome, 46,XX GD, or AD yet. We thought that the etiology of dysplasia of the female reproductive system is related to the toxicant exposure of her parents during the gestation period. A number of toxicants, such as phthalate esters (PEs), have been reported to have a direct link with GD when exposed during early pregnancy (16). Animal studies have confirmed that *utero* exposure of PEs lead to characteristics of MRKH syndrome in SD rats (17). Moreover, long-term ambient fine particles (PM2.5) exposure could induce ovarian dysfunction in mice (18). Therefore, environmental pollution plays an important role in the dysgenesis of the reproductive system, but the mechanism remains to be explored.

## Data availability statement

The datasets presented in this study can be found in online repositories. The names of the repository/repositories and accession number(s) can be found in the article/supplementary material.

## Ethics statement

The studies involving human participants were reviewed and approved by the Second Xiangya Hospital, Central South University. The patients/participants provided their written informed consent to participate in this study. Written informed consent was obtained from the individual(s) for the publication of any potentially identifiable images or data included in this article.

## Author contributions

YZ drafted the manuscript. HT and YH designed the study. HT and LT revised the manuscript. BJ, YZ, and HT were responsible for the collection of data or analysis. All authors contributed to the article and approved the submitted version.

## Funding

This work was supported by the Key Research and Development Program of Hunan Province of China [Award number(s): 2019SK2022].

## Conflict of interest

The authors declare that the research was conducted in the absence of any commercial or financial relationships that could be construed as a potential conflict of interest.

## Publisher's note

All claims expressed in this article are solely those of the authors and do not necessarily represent those of their affiliated organizations, or those of the publisher, the editors and the reviewers. Any product that may be evaluated in this article, or claim that may be made by its manufacturer, is not guaranteed or endorsed by the publisher.
